# Serum Levels of Adipocyte Fatty Acid-Binding Protein Are Associated with the Severity of Coronary Artery Disease in Chinese Women

**DOI:** 10.1371/journal.pone.0019115

**Published:** 2011-04-28

**Authors:** Yuqian Bao, Zhigang Lu, Mi Zhou, Huating Li, Ye Wang, Meifang Gao, Meng Wei, Weiping Jia

**Affiliations:** 1 Department of Endocrinology and Metabolism, Shanghai Jiao Tong University Affiliated Sixth People's Hospital and Shanghai Diabetes Institute, Shanghai Key Laboratory of Diabetes Mellitus, Shanghai Clinical Center for Diabetes, Shanghai, China; 2 Department of Cardiology, Shanghai Jiao Tong University Affiliated Sixth People's Hospital, Shanghai, China; 3 Department of Medicine, Shanghai Jiao Tong University School of Medicine, Shanghai, China,; University of Hong Kong, China

## Abstract

**Background:**

Adipocyte fatty acid-binding protein (A-FABP) has been described as a novel adipokine, playing an important role in the development of metabolic syndrome, type 2 diabetes and atherosclerosis. In this study, we investigated the relationship between serum levels of A-FABP and the presence and severity of coronary artery disease (CAD) in Chinese subjects.

**Methodology/Principal Findings:**

Circulating A-FABP level was determined by ELISA in 341 Chinese subjects (221 men, 120 women) who underwent coronary angiography. A-FABP levels in patients with CAD were significantly higher compared with non-CAD subjects (*P* = 0.029 in men; *P* = 0.031 in women). Serum A-FABP increased significantly in multi-vessel diseased patients than in non-CAD subjects (*P* = 0.011 in men, *P* = 0.004 in women), and showed an independent correlation with coronary atherosclerosis index (standardized β = 0.173, *P* = 0.025). In multiple logistic regression analysis, serum A-FABP was an independent risk factor for CAD in women (OR = 5.637, 95%CI: 1.299-24.457, *P* = 0.021). In addition, amino terminal pro-brain natriuretic peptide (NT-proBNP) was demonstrated to be positively and independently correlated with A-FABP (standardized β = 0.135, *P* = 0.027).

**Conclusions/Significance:**

Serum A-FABP is closely associated with the presence and severity of CAD in Chinese women.

## Introduction

Adipose tissue functions as an endocrine organ, secreting a variety of bioactive substances (called adipokines), which are actively involved in insulin sensitivity, glucose and lipid metabolism [1]. Adipocyte fatty acid-binding protein (A-FABP, also known as ap2 and FABP4), a member of the fatty acid binding protein super family, has recently been described as a novel adipokine, accounting for approximately 6% of total cellular proteins in mature adipocytes [2]. It is also present in macrophages, which possess similar functions as adipocytes, and modulated by proliferator-activated receptor-γ agonists and oxidized low density lipoproteins [3].

There is substantial experimental evidence that A-FABP plays an important role in metabolic deterioration and the development of atherosclerosis [3]. Mice deficient in A-FABP were protected from development of insulin resistance, hyperglycemia and dyslipidemia associated with genetic or diet-induced obesity [4], [5]. A recent study from Furuhashi et al. demonstrated that A-FABP exerted actions in both adipocytes and macrophages, and the interactions between these two cell types were critical for the impact of A-FABP on metabolic deterioration [6]. In apolipoprotein E-deficient (apo E^-/-^) mice, A-FABP deficiency reduced early foam cell formation greatly, and provided remarkable protection against atherosclerosis with significant reductions in mean atherosclerotic lesion area on a chow diet or a western diet independent of the effects of A-FABP on insulin resistance and plasma lipids [7], [8]. Moreover, an orally active A-FABP inhibitor can remarkably prevent atherosclerosis in apo E^-/-^ mice [9]. Similar to A-FABP deficiency in experimental models, a genetic variant at A-FABP locus with reduction in A-FABP expression reduced the risk for cardiovascular disease and type 2 diabetes in a population genetic study [10].

Although originally identified as a major cytoplasmic protein, A-FABP is also released from adipose tissue into the circulation [11]. In keeping with the experimental findings, several clinical investigations showed that serum A-FABP was associated with the parameters of insulin resistance, adiposity, and hyperglycemia [11]. Serum A-FABP levels were found to be independently associated with carotid atherosclerosis in Chinese women [12]. Recent two studies in Korean adults and Chinese found that A-FABP levels tended to be higher in CAD patients compared to non-CAD subjects (*P* = 0.096 and *P* = 0.088) [13], [14]. Furthermore, both studies have demonstrated significantly higher serum A-FABP levels in subjects with 3-vessel disease than those without CAD and those with 1-vessel disease. These observations from animal models and human suggest A-FABP may be an important player in the development of CAD. Therefore, this study was conducted to further explore the role of A-FABP in CAD, and to determine whether A-FABP influences CAD independent of the traditional risk factors. In addition, we also measured high sensitivity C-reactive protein (hsCRP) and amino terminal pro-brain natriuretic peptide (NT-proBNP), two biomarkers involved in cardiovascular disease, to investigate the possible mechanisms mediating the impact of A-FABP on the development of CAD.

## Methods

### Ethics Statement

All subjects gave written informed consent, and the study was approved by the ethics committee of Shanghai Jiao Tong University Affiliated Sixth People's Hospital and complied with the Declaration of Helsinki.

### Participants

The study population consisted of 341 participants (221 men and 120 women, aged 66.1±10.4 years) who were admitted to the Department of Cardiology of Shanghai Jiao Tong University Affiliated Sixth People's Hospital to undergo coronary angiography between July 2008 and October 2009. All women were postmenopausal. The diagnosis of type 2 diabetes was based on 1999 World Health Organization criteria [15]. According to the definition of Chinese Joint Committee for Developing Chinese Guidelines on Prevention and Treatment of Dyslipidemia in Adults [16], the metabolic syndrome was defined as having ≥3 of the following metabolic risk factors: (1) central obesity (waist circumference >90 cm for men and >85 cm for women), (2) fasting triglycerides (TG) ≥1.70 mmol/l, (3) fasting high density lipoprotein cholesterol (HDL-c) < 1.04 mmol/l, (4) hypertension (sitting blood pressure ≥130/85 mm Hg or known treatment for hypertension), (5) hyperglycemia defined as fasting glucose (FPG) ≥6.1 mmol/l and/or 2-h postchallenge glycemia (2hPG) ≥7.8 mmol/l or on hypoglycemic therapy for treatment of diabetes. Medical history, medication history and smoking habits were assessed using a standardized questionnaire. Those who had regularly smoked in the past or who smoked at least one cigarette per day lasting for at least one year were included into the analysis.

Subjects with the following conditions were excluded: hepatic or renal dysfunction, recent myocardial infarction (<3 months), previous coronary by-pass surgery or percutaneous coronary intervention (<6 months), congestive heart failure (New York Heart Association Class III∼IV), acute infection, and history of malignancy.

### Coronary angiography

Coronary angiography was performed using standard Judkins techniques [17], and all major coronary arteries were carefully imaged in at least two orthogonal views. Angiographic analysis was carried out by 2 experienced interventional cardiologists, who were unaware of clinical information of the patients. CAD was defined as the presence of 50% or greater coronary diameter lumen stenosis in a major coronary artery (left main coronary artery, left anterior descending artery or its first diagonal branch, left circumflex artery or its first obtuse marginal branch, and right coronary artery). According to the number of diseased vessels, CAD patients were further divided into 1-vessel and multi-vessel disease groups which included patients with 2-vessel and 3-vessel disease. Left main stenosis ≥50% was considered as 2-vessel disease.

The extent and severity of CAD was assessed by assigning points to each lesion as follows: no significant stenosis of the luminal diameter, 0; stenosis of less than 25%, 1; 25–49% stenosis, 2; 50–74%, 3; 75–100%, 4. The coronary atherosclerosis index (CAI) was defined as the sum of these scores [18].

### Anthropometric evaluation

A complete physical examination was performed on each subject. Body mass index (BMI) was calculated as weight in kilograms divided by squared height in meters (kg/m^2^). Waist circumference was measured midway between the lowest rib and the superior border of iliac crest on midaxillary line.

### Biochemical assessment

Blood samples were collected after an overnight fast and stored at −80°C prior to analysis. Subjects without diabetes received a standard 75 g oral glucose tolerance test. FPG and 2hPG were determined using glucose oxidase method. Serum insulin was assayed via radio-immunoassay (Linco Research, St Charles, Missouri, USA). Glycated hemoglobin A1c (HbA1c) values were measured by high performance liquid chromatography (Bio-Rad Laboratories, Hercules, CA, USA). Serum creatinine and lipid profiles, including total cholesterol (TC), TG, HDL-c and low density lipoprotein cholesterol (LDL-c) were determined by enzymatic procedures using an autoanalyser (Hitachi 7600–020; Hitachi, Tokyo, Japan). Serum hsCRP was measured by a particle-enhanced immunoturbidimetric assay (Dade Behring Inc., Newark, USA). NT-proBNP was assayed by chemiluminescence (Cobas 6000, Roche Diagnostics GmbH, Mannheim, Germany). A-FABP levels were assayed by sandwich ELISA (BioVendor Laboratory Medicine, Modrice, Czech Republic). Insulin resistance was estimated using homeostasis model assessment index (HOMA-IR) [19]. Renal function was evaluated by the estimated glomerular filtration rate (GFR) derived from Cockroft-Gault formula [20].

### Statistical analysis

All analyses were performed using SPSS version 13.0 (SPSS, Chicago, IL). Data were reported as means ± SD for normal distributions or median with interquartile range for skewed variables. Data that were not normally distributed as determined by the Kolmogorov-Smirnov test were logarithmically transformed before analysis. Characteristics of subjects between non-CAD and CAD groups or men and women were compared using unpaired Student's t-test (for data that were normally distributed) or the Mann-Whitney U-test (not normally distributed). One-way ANOVA was used to compare serum A-FABP levels in groups according to number of diseased vessels. The chi-square test was used to compare categorical variables between groups. The Spearman correlation analysis was used to assess the correlations of CAI and serum A-FABP with metabolic parameters. To determine the independent parameters correlated with CAI and A-FABP, parameters including age, gender, and those correlated significantly with CAI and A-FABP in correlation analysis were tested in multiple stepwise regression analysis. Multiple logistic regression analysis was performed to identify independent predictors for CAD. Parameters that significantly different between subjects with and without CAD and conventional risk factors including lipid profile (TC, TG, LDL-c, HDL-c) were selected to enter into multiple logistic regression. Two-sided values of *P*<0.05 were considered as statistically significant.

## Results

The clinical characteristics of the subjects are shown in Table 1. Of the 341 subjects, 94 were without CAD (46 men, 48 women), while 247 suffered from CAD (175 men, 72 women). Patients with CAD had higher age, HbA1c, NT-proBNP, CAI and the proportion of statin use, as well as lower GFR compared with those without CAD in both genders (all *P*<0.05). Women with CAD also had significant higher systolic blood pressure, 2hPG and the percentage of hypertension than non-CAD controls (all *P*<0.05).

**Table 1:** Characteristics of the subjects according to the presence or absence of CAD.

	Men		Women	
	Non-CAD (n = 46)	CAD(n = 175)	Non-CAD (n = 48)	CAD (n = 72)
Age (years)	60.6±10.4	66.5±10.4 b	64.8±10.2	69.4±9.4 a
BMI (kg/m^2^)	24.4±4.1	24.5±3.2	25.1±4.2	24.2±2.9
Waist circumference (cm)	91.9±11.3	91.4±9.1	86.3±10.1	88.6±8.2
Systolic BP (mmHg)	132.6±20.9	133.9±18.5	130.6±15.8	138.1±18.2 a
Diastolic BP (mmHg)	80.4±10.6	77.9±10.6	78.3±10.3	77.1±10.9
FPG (mmol/l)	5.6±1.0	5.9±1.4	5.9±1.7	6.2±1.7
2hPG (mmol/l)	9.5±3.9	9.9±4.2	8.2±3.9	9.8±3.7 a
Fasting insulin (mU/l)	17.0±6.2	25.6±10.3	20.1±9.0	24.1±10.6
HbA1c (%)	6.0 (5.6–6.7)	6.2 (5.8–7.0) a	6.0 (5.7–6.4)	6.4 (5.9–7.1) a
HOMA-IR	3.8 (3.2–5.7)	4.2 (3.3–6.6)	4.9 (2.7–6.7)	4.8 (3.4–6.9)
TC (mmol/l)	4.2±1.0	4.1±1.0	4.9±1.0	4.5±1.2
TG (mmol/l)	1.4 (1.1–2.2)	1.5 (1.0–2.0)	1.4 (0.8–2.3)	1.6 (1.2–2.6)
HDL-c (mmol/l)	1.0 (0.8–1.2)	1.0 (0.8–1.2)	1.3 (1.1–1.5)	1.2 (0.9–1.3)
LDL-c (mmol/l)	2.9±1.0	2.7±0.9	3.4±0.9	3.0±1.1
Creatinine (µmmol/l)	84 (71–93)	84 (75–96)	60 (56–71)	65 (56–77)
GFR (ml/min)	105.5 (84.7–145.1)	93.7 (76.4–114.1) a	115.2 (84.3–128.4)	97.4 (77.2–127.6) a
hsCRP (mg/l)	1.9 (0.6–4.6)	1.4 (0.7–4.6)	1.5 (0.3–2.4)	1.5 (0.5–3.5)
NT-proBNP (ng/l)	105.5 (51.8–222.7)	126.2 (62.4–399.2) a	90.7 (42.6–305.4)	161.3 (94.6–546.0) a
CAI	0.0 (0.0–1.0)	10.0 (6.0–15.0) b	0 (0–0.8)	8 (4.3–13.8) b
Metabolic syndrome (%)	64.3	60.1	54.5	66.2
Central obesity (%)	48.8	59.2	57.8	64.3
Hyperglycemia (%)	67.4	64.0	50.0	61.1
Hypertriglyceridemia (%)	44.4	38.5	48.9	47.1
Low HDL-c (%)	55.6	57.4	23.4	37.1
Hypertension (%)	82.6	77.1	70.8	86.1 a
Smoking (%)	76.1	65.1	2.1	2.8
Statins use (%)	8.7	32.6 b	12.5	31.9 a

Data are means ± SD or median (interquartile range). BMI: body mass index; BP, blood pressure; CAI: coronary atherosclerosis index; FPG: fasting plasma glucose; 2hPG: 2-h postchallenge glycemia; GFR: glomerular filtration rate; HbA1c: glycated hemoglobin A1c; HDL-c: high density lipoprotein cholesterol; HOMA-IR: homeostasis model assessment index of insulin resistance; hsCRP: high sensitivity C-reactive protein; LDL-c: low density lipoprotein cholesterol; NT-proBNP: amino terminal pro-brain natriuretic peptide; TC: total cholesterol; TG: triglyceride.

a *P*<0.05 vs. Non-CAD.

b *P*<0.01 vs. Non-CAD.

**Figure 1:**
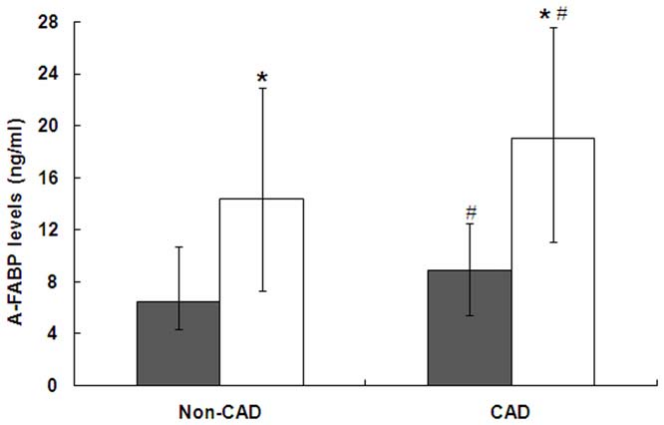
Serum A-FABP levels in subjects with and without CAD. Data are median with interquartile range. Black, men; white, women. * *P*<0.001 versus men. # *P*<0.05 versus non-CAD group.

As shown in Figure 1, serum A-FABP levels were significantly higher in women than in men in both CAD group (19.0 ng/ml (11.7–25.9) vs. 8.9 ng/ml (5.3–12.5), *P*<0.001) and non-CAD group (14.4 ng/ml (7.4–22.9) vs. 6.5 ng/ml (4.4–10.7), *P*<0.001). Also, A-FABP levels in patients with CAD increased significantly compared with non-CAD subjects in both men and women (*P* = 0.029 and *P* = 0.031, respectively).

**Figure 2:**
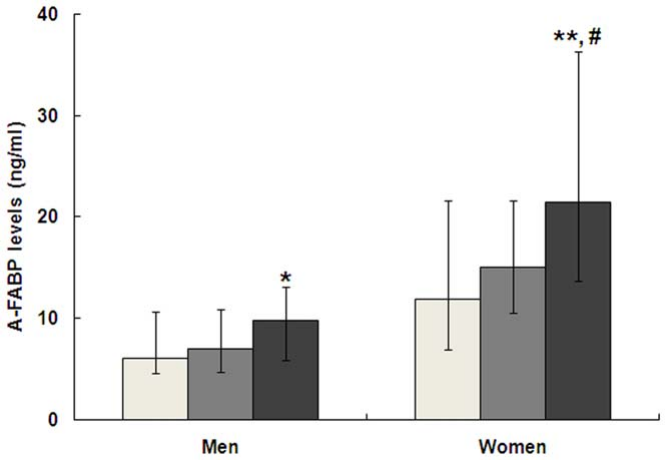
Comparison of serum A-FABP levels among groups with different numbers of diseased vessels. * *P*<0.05, ** *P*<0.01 versus non-CAD group. # *P*<0.05 versus 1 vessel diseased group. White, non-CAD group; Grey, 1 vessel diseased group; Black, multi-vessel diseased group. The median A-FABP levels were 7.2 ng/ml (4.5–11.5) and 9.8 ng/ml (5.9–14.0) for men with 1 and multi-vessel disease (n = 56 and 119), and 15.0 ng/ml (10.5–21.7) and 21.43 ng/ml (16.0–36.8) for women (n = 35 and 37), respectively.

We next analyzed the relationship between serum A-FABP and the severity of CAD. In all subjects, CAI was positively associated with age (r = 0.371, *P*<0.001). After adjusting for age and gender, CAI showed a positive correlation with HbA1c, fasting insulin, HOMA-IR, NT-proBNP and serum A-FABP (*P*<0.05), and an inverse correlation with HDL-c (*P* = 0.029) (Table 2). In multiple stepwise regression analysis after controlling for age, gender, HOMA-IR, HbA1c, and HDL-c, A-FABP was shown to be independently correlated with CAI (standardized β = 0.173, *P* = 0.025). Furthermore, as shown in Figure 2, the serum levels of A-FABP increased progressively with the increasing number of diseased vessels (*P* = 0.008 in men and *P* = 0.004 in women). Compared with those without CAD, patients with multi-vessel disease had significantly higher serum A-FABP levels in both men (*P* = 0.011) and women (*P* = 0.004). In addition, significant higher levels of A-FABP were also observed in women with multi-vessel disease compared with those with 1 vessel disease (*P* = 0.031).

**Table 2:** Correlation of CAI with cardiometabolic risk factors.

	CAI (age and gender adjusted)	
	r	*P*
BMI	0.044	NS
Waist circumference	−0.049	NS
Systolic BP	0.074	NS
Diastolic BP	−0.026	NS
FPG	0.084	NS
2hPG	0.096	NS
HbA1c	0.145	0.021
Fasting insulin	0.149	0.020
HOMA-IR	0.147	0.023
TC	−0.003	NS
TG	0.050	NS
HDL-c	−0.137	0.029
LDL-c	−0.004	NS
hsCRP	0.084	NS
NT-proBNP	0.237	0.001
A-FABP	0.134	0.032
Smoking	0.036	NS

A-FABP: Adipocyte fatty acid-binding protein; BMI: body mass index; BP, blood pressure; CAI: coronary atherosclerosis index; FPG: fasting plasma glucose; 2hPG: 2-h postchallenge glycemia; HbA1c: glycated hemoglobin A1c; HDL-c: high density lipoprotein cholesterol; HOMA-IR: homeostasis model assessment index of insulin resistance; hsCRP: high sensitivity C-reactive protein; LDL-c: low density lipoprotein cholesterol; NT-proBNP: amino terminal pro-brain natriuretic peptide; TC: total cholesterol; TG: triglyceride.

NS, Not significant.

To evaluate the relationship between A-FABP and CAD, multiple logistic regression analysis was performed using the presence of CAD as a dependent variable (Table 3). The analysis involved age, systolic blood pressure, 2hPG, HbA1c, lipid profile (TC, TG, LDL-c, HDL-c), GFR, NT-proBNP and A-FABP. As a result, serum A-FABP (OR = 5.637, 95%CI: 1.299–24.457, *P* = 0.021) and systolic blood pressure (OR = 1.075, 95%CI: 1.017–1.137, *P* = 0.010) were demonstrated to be independent factors for CAD in women. However in men, only age was independently associated with the presence of CAD (OR = 1.066, 95%CI: 1.007–1.128, *P* = 0.029).

**Table 3:** Multivariate logistic regression analysis showing the parameters independently associated with CAD.

	B	S.E.	P	OR	95% CI
Women only (n = 120)					
A-FABP a	1.729	0.749	0.021	5.637	1.299–24.457
Systolic BP	0.073	0.028	0.010	1.075	1.017–1.137
Men only (n = 221)					
Age	0.064	0.029	0.029	1.066	1.007–1.128

a Logarithmically transformed before analysis.

Variables included in the original model are age, systolic BP, 2hPG, HbA1c, TC, TG, LDL-c, HDL-c, GFR, NT-proBNP and A-FABP.

A-FABP showed significant positive correlations with age, BMI, waist circumference, 2hPG, HOMA-IR, creatinine, hsCRP, and NT-proBNP, and a negative correlation with GFR in both genders (all *P*<0.05) (Table 4). A-FABP also correlated with FPG, HbA1c, fasting insulin, and HDL-c in men as well as LDL-c in women (all *P*<0.05). To further determine which variables were independently correlated with serum A-FABP, multiple stepwise regression analysis was performed in all subjects (Table 5). The analysis involved age, gender, waist circumference, FPG, 2hPG, HbA1c, HOMA-IR, lipid profile, GFR, hsCRP, and NT-proBNP. As a result, gender (women), waist circumference, HbA1c, HOMA-IR, LDL-c, GFR and NT-proBNP were independently correlated with A-FABP (all *P*<0.05).

**Table 4:** Correlations of A-FABP levels with anthropometric parameters and biochemical indexes.

	All		Men		Women	
	r	*P*	r	*P*	r	*P*
Age	0.371	<0.001	0.325	<0.001	0.260	0.005
BMI	0.163	0.003	0.139	0.042	0.341	<0.001
Waist circumference	0.161	0.004	0.215	0.002	0.406	<0.001
Systolic BP	0.057	NS	−0.008	NS	0.127	NS
Diastolic BP	−0.016	NS	−0.039	NS	0.120	NS
FPG	0.152	0.005	0.142	0.036	0.155	NS
2hPG	0.127	0.022	0.161	0.020	0.222	0.018
HbA1c	0.214	<0.001	0.313	<0.001	0.163	NS
Fasting insulin	0.228	<0.001	0.239	0.001	0.148	NS
HOMA-IR	0.240	<0.001	0.333	<0.001	0.404	<0.001
TC	0.118	0.033	−0.089	NS	0.152	NS
TG	0.077	NS	−0.006	NS	0.131	NS
HDL-c	0.029	NS	−0.163	0.018	−0.021	NS
LDL-c	0.143	0.010	−0.013	NS	0.218	0.019
Creatinine	0.171	0.002	0.436	<0.001	0.409	<0.001
GFR	−0.294	<0.001	−0.370	<0.001	−0.298	0.002
hsCRP	0.168	0.002	0.136	0.048	0.298	0.001
NT-proBNP	0.278	<0.001	0.280	<0.001	0.242	0.022

BMI: body mass index; BP, blood pressure; FPG: fasting plasma glucose; 2hPG: 2-h postchallenge glycemia; GFR: glomerular filtration rate; HbA1c: glycated hemoglobin A1c; HDL-c: high density lipoprotein cholesterol; HOMA-IR: homeostasis model assessment index of insulin resistance; hsCRP: high sensitivity C-reactive protein; LDL-c: low density lipoprotein cholesterol; NT-proBNP: amino terminal pro-brain natriuretic peptide; TC: total cholesterol; TG: triglyceride.

NS, Not significant.

**Table 5:** Multiple stepwise regression analysis showing variables independently associated with serum A-FABP.

Independent variables	β	S.E.	Standardized β	*P*
Gender (women)	0.311	0.038	0.460	<0.001
Waist circumference	0.007	0.002	0.211	0.001
HbA1c a	0.044	0.018	0.141	0.017
HOMA-IR a	0.194	0.078	0.145	0.013
LDL-c	0.044	0.018	0.135	0.017
GFR a	−0.574	0.116	−0.309	<0.001
NT-proBNP a	0.070	0.031	0.135	0.027

Variables included in the original model are age, gender, waist circumference, FPG, 2hPG, HbA1c, HOMA-IR, lipid profile, GFR, hsCRP, and NT-proBNP.

a Logarithmically transformed before analysis.

GFR: glomerular filtration rate; HbA1c: glycated hemoglobin A1c; HOMA-IR: homeostasis model assessment index of insulin resistance; LDL-c: low density lipoprotein cholesterol; NT-proBNP: amino terminal pro-brain natriuretic peptide.

## Discussion

In the current study, we provided the clinical evidence showing that A-FABP was closely associated with the severity of coronary atherosclerosis, and was a significant risk factor for the development of CAD in women. Furthermore, we found a close relationship of serum A-FABP with NT-proBNP in both genders.

The relationship of A-FABP with metabolic disease has been well demonstrated in several clinical studies, which might partly explain the impact of A-FABP on CAD. Xu et al. demonstrated that A-FABP levels were not only associated with insulin resistance, adiposity, dyslipidemia and glucose tolerance parameters, but also predicted the development of the metabolic syndrome and type 2 diabetes in 5-year and 10-year prospective studies [11], [21], [22]. In agreement with these findings, we found that waist circumference, HbA1c, LDL-c and HOMA-IR were independently associated with serum A-FABP. The mechanism of these associations has been investigated in several studies. A-FABP is capable of binding to various intracellular hydrophobic compounds such as saturated and unsaturated long-chain fatty acids, modulating cholesterol ester accumulation, and mediating intracellular lipid trafficking, thus altering cellular and systemic lipid transport and composition, as well as contributing to dyslipidemia [23], [24]. In skeletal muscle, insulin receptor phosphorylation and AMP-activated kinase activity were found to be enhanced in A-FABP deficient models, thus resulting in enhanced insulin signaling and fatty acid oxidation [23]. Insulin resistance has been verified to be closely associated with atherosclerosis, and the metabolic syndrome is being increasingly recognized to be an important risk factor for cardiovascular disease. An 11-year prospective study in a population-based cohort of middle-aged Finnish men suggested that the metabolic syndrome was associated with a nearly 3 fold higher mortality from CAD [25]. Therefore, the association of serum A-FABP with CAD might be partly attributed to the effect of A-FABP on metabolic deterioration.

In addition to its metabolic actions, A-FABP also plays a critical role in the development of atherosclerosis through coordinating macrophage cholesterol trafficking and inflammatory activity [26]. High levels of A-FABP were found in atherosclerotic lesions in both mice and humans [26]. In the absence of A-FABP, cholesterol trafficking was enhanced [26] and the capacity for foam cell formation of macrophages was reduced significantly [7]. In addition, A-FABP can activate several key inflammatory pathways. In A-FABP deficient macrophages, the IκB kinase –NF-κB pathway is impaired, resulting in suppression of inflammatory function. Besides that, peroxisome proliferator-activated receptor γ and the liver X receptor α activity are enhanced, leading to the suppressed transcription of several inflammatory genes [27], [28]. BMS309403, a specific A-FABP inhibitor, was found to have remarkable impact on the inhibition of atherosclerosis in the apo E^-/-^ mouse model on a western diet [9]. In clinical studies, Yeung et al. found that serum A-FABP was an independent determinant of carotid atherosclerosis as determined by carotid intima-media thickness in Chinese women [12]. Miyoshi et al reported that A-FABP was independently associated with coronary plaque burden measured by intravascular ultrasound in 125 CAD patients, and women had a greater impact than in men [29]. Similarly in the present study, A-FABP not only significantly elevated in CAD patients, but was closely associated with the presence of CAD in women, independent of the influence of the traditional risk factors. We further found a positive correlation of serum A-FABP with the severity of coronary atherosclerosis. Serum A-FABP concentration increased significantly with the increasing number of stenotic vessels, which was in accordance with the recent findings from Rhee et al. and Jin et al [13], [14]. Taken together, these observations suggest that serum A-FABP may be a new marker for CAD and its circulating levels may predict the extent and severity of coronary atherosclerosis in women.

Another notable observation of this study is the independent association between serum A-FABP and NT-proBNP, a hormone secreted by cardiomyocytes following increased myocardial wall stress and exerts favorable effects on heart such as promoting vasodialation [30]. Circulating concentrations of NT-proBNP have been used for the diagnosis/prognosis of heart failure. An increasing body of evidence also suggests that myocardial ischemia is a potent stimulus for BNP release, and NT-proBNP is a promising biomarker for evaluating and predicting myocardial ischemia in CAD [31]. A recent study reported that A-FABP directly and acutely depressed contraction of cardiomyocytes by attenuating intracellular Ca^2+^ levels [32], suggesting a direct bioactive role of A-FABP may exist in heart function. Therefore, the elevated A-FABP may have an impact on the myocardial synthesis of BNP through inducing coronary atherosclerosis or directly influencing myocardial function. However, the mechanism underlying the relationship between A-FABP and NT-proBNP is not clear and remain to be further elucidated. Furthermore, we also observed a positive association between serum A-FABP and hsCRP. HsCRP is a well-known inflammatory biomarker that can predict future cardiovascular events [33]. It plays a direct pathogenic role in atherosclerosis by inducing expression of nuclear factor-κB and adhesion molecules, stimulating macrophages to produce tissue factor, increasing the expression and activity of plasminogen activator inhibitor-1 in endothelia cells [34], [35]. Therefore, our finding provided further evidence of serum A-FABP in contributing to inflammatory activity and atherosclerosis.

In the present study, we also found an independent negative relationship between A-FABP and renal function as assessed by GFR and serum creatinine, which is in accordance with a recent study [36]. These observations support the notion that renal filtration is a major route of elimination of A-FABP, which is similar in several other adipokines like adiponectin and leptin.

This study has several limitations. First, the sample size was not large enough. Second, the present study is cross-sectional, which does not allow us to determine the cause-effect relationship between A-FABP and the development of CAD. Further large population-based prospective studies are needed to address whether serum A-FABP is useful for predicting CAD. Furthermore, in order to obtain more accurate data about coronary stenosis, we only recruited subjects admitted to hospital to undergo coronary angiography, and most of these people have existing risk factors of CAD, or already on intervention therapy. Whether our findings can be replicated in the general population still needs to be further confirmed.

In conclusion, we demonstrated that serum A-FABP was closely associated with the presence and severity of CAD in Chinese women. A-FABP may be an independent marker and predictor of CAD. Our data, in conjunction with previous animal and clinical studies, support the central role of A-FABP in the development of the metabolic syndrome, diabetes and cardiovascular disease. Further studies are warranted to evaluate whether A-FABP can be used to predict the risk of CAD in larger study cohorts recruited from the general population.
